# The Effectiveness of Social Media Campaigns in Improving Knowledge and Attitudes Toward Mental Health and Help-Seeking in High-Income Countries: Scoping Review

**DOI:** 10.2196/68124

**Published:** 2025-05-23

**Authors:** Ruth Plackett, Jessica-Mae Steward, Angelos P Kassianos, Marvin Duenger, Patricia Schartau, Jessica Sheringham, Silvie Cooper, Lucy Biddle, Judi Kidger, Kate Walters

**Affiliations:** 1 Department of Primary Care and Population Health University College London London United Kingdom; 2 Department of Nursing Cyprus University of Technology Limassol Cyprus; 3 UCL Medical School University College London London United Kingdom; 4 Bristol Medical School Population Health Sciences University of Bristol Bristol United Kingdom

**Keywords:** social media, health campaigns, social marketing, review, mental health, public health, PRISMA

## Abstract

**Background:**

The prevalence of mental health problems is increasing, particularly among young people, making the prevention of mental health problems and improvements in care a public health priority. Social media, with its wide reach and low-cost information dissemination, has emerged as an important tool for public mental health campaigns in high-income countries. However, there is a limited understanding of the reach of public mental health social media campaigns and their impact on mental health knowledge, attitudes, stigma, and behaviors, such as help-seeking.

**Objective:**

This review aimed to assess the effectiveness of social media campaigns in high-income countries in improving knowledge and attitudes toward mental health, reducing stigma, promoting help-seeking behavior, and reaching underserved communities.

**Methods:**

A scoping review was conducted, involving a comprehensive search of 5 databases (MEDLINE, Embase, PsycINFO, Web of Science, and CINAHL) and gray literature from January 2004 to May 2024. We included quantitative evaluations of social media public mental health campaigns from high-income countries with comparable social media use and public health care systems. A narrative synthesis summarized the study characteristics, campaign exposure, reach, and effectiveness by outcomes.

**Results:**

The review included 26 studies for analysis. Less than half of the studies (11/26, 42%) reported on the reach of mental health campaigns, but for those that did, younger age groups and women were more likely to be aware of campaigns. The most frequently reported outcomes were attitudes about mental health (17/23, 74%) and stigma (17/23, 74%), followed by mental health knowledge (16/23, 70%) and behavior change (15/23, 65%), such as seeking help for a mental health condition. While stigma and attitudes showed the most improvement before and after the campaigns (5/11, 45%), behavior change showed the least positive change over time (1/8, 13%). However, behavior change was the most improved outcome for those who were campaign aware compared to those unaware (12/12, 100%), whereas positive attitudes often did not differ. In fact, some studies showed campaign awareness was associated with negative stereotypes.

**Conclusions:**

The evidence highlights the potential of social media campaigns in improving mental health knowledge, attitudes, stigma, and behavior change. However, due to the methodological limitations of these evaluations, it is challenging to determine if the positive changes in these outcomes are a result of the campaigns or other factors. Campaign awareness seems to be important for initiating behavior change, but these changes are often short-lived. Sustainable impact on mental health requires both individual behavior change and service improvements. Targeting more mental health campaigns at underserved groups could help to reduce stigma and raise awareness in these groups, which could lead to timelier access to services. Consistent measurement of campaign reach and behavior change outcomes could help to understand and maximize campaign impact.

## Introduction

The prevalence of mental disorders has increased in the last 20 years, particularly for young people, and preventing and addressing poor mental health has become a public health priority for many countries [1-5]. Data show that increasing numbers of people are seeking help from health care services for their mental health concerns [6-8]. However, many people still do not seek help for mental health problems, in part due to stigma, poor access, and lengthy waiting lists [9-11]. It is important to seek appropriate and timely help for mental health problems, as early detection of issues can reduce the risk of experiencing further mental health problems and complications associated with mental illness. Early intervention can also reduce costs in the use of health services because of better mental health [9].

Social media has the potential to be an effective tool in public mental health campaigns to reduce stigma, raise awareness of mental health, encourage help-seeking, and access to mental health care, as it can disseminate information quickly to a wide audience at a low cost [12]. Social media refers to “internet-based tools that allow individuals and communities to gather and communicate; to share information, ideas, personal messages, images, and other content; and, in some cases, to collaborate with other users in real time” [13,14]. Social media use has become ubiquitous; it is estimated that 5.17 billion people globally use at least one form of social media in 2024 [15]. Given the number of people using social media, there is great potential to reach large numbers of people with communications about mental health awareness and services and address stigma regarding mental health [12,16]. Social media may also be more likely than traditional campaigns to capture the attention of and reach certain underserved groups who frequently use social media [17-19]. These groups, such as LGBTQ+ (lesbian, gay, bisexual, transgender, queer/questioning, and others) young people, are important to reach because they are disproportionately affected by mental health problems and often face challenges in accessing care [17,20-23].

Behavior change theories, such as the Capability, Opportunity, Motivation, and Behavior Change (COM-B) model, provide a framework for understanding how social media public health campaigns can result in behavior change, such as increased seeking of mental health services [16,24,25]. This model argues that changes in behavior occur as the result of an interaction between capability, opportunity, and motivation. Social media public mental health campaigns can improve people’s psychological capabilities to engage in behaviors like help-seeking by improving mental health awareness, knowledge, literacy, and capacity to navigate mental health service access. They can improve people’s motivation to engage in behaviors by changing reflective motivations or attitudes about mental health problems and discouraging fear of mental ill health. Campaigns can also improve opportunities for behavior change by influencing social and cultural norms and stigma around mental health and providing access to resources. These campaigns must also effectively reach and engage individuals to facilitate changes in their knowledge, attitudes, and behaviors [26].

There are no previous reviews that have synthesized the evidence for how effective social media public mental health campaigns are at changing behaviors like help-seeking for mental health concerns. Further, none have examined how effective they are at facilitating the components of this behavior change, such as improving knowledge, changing attitudes, and reducing stigma. There is also limited understanding of whether social media campaigns reduce inequalities in access and help-seeking by reaching underserved groups for mental health problems, such as those from ethnic minority groups, LGBTQ+ individuals, those who are socioeconomically disadvantaged, and young men [20,22]. Given the lack of previous reviews and the increasing use of social media in public health campaigns, there is a need for a broader mapping of the extent of the evidence relating to public mental health social media campaigns [27]. Understanding the impact of social media campaigns on behavior change, including knowledge, attitudes, and stigma, especially for underserved groups, could help improve targeted help-seeking campaigns and identify gaps in the literature that need further investigation. This review will focus on high-income countries where the use and access to social media and resources for public health campaigns are comparable. The review questions include the following: (1) Is there evidence that social media campaigns have changed behavior (eg, led to increased help-seeking), improved knowledge and attitudes toward mental health, and reduced stigma in high-income countries? (2) To what extent are social media campaigns effective at reaching underserved groups and changing their behaviors, knowledge, attitudes and reducing stigma in high-income countries?

## Methods

### Study Design

We developed a review protocol according to the methodological guidance for scoping reviews [27-29], and the protocol is available via the Open Science Framework [30]. This review is reported according to PRISMA-ScR (Preferred Reporting Items for Systematic Reviews and Meta-Analyses extension for Scoping Reviews; Multimedia Appendix 1) guidelines [31].

### Search Strategy

An experienced research librarian helped to develop the search strategy, which included a combination of subject headings and keyword searches for the key concepts of “Social Media,” “Mental Health,” “Health Knowledge,” “Attitudes,” “Stigma,” and “Help Seeking Behavior.” A total of 5 databases were searched, including MEDLINE, Embase, PsycINFO, Web of Science, and CINAHL. Multimedia Appendix 2 shows the full search strategy for MEDLINE, which was adapted for the other databases. The gray literature, such as public mental health campaign websites identified from the review and leading mental health charity websites in the United Kingdom and other high-income countries, were searched for relevant reports not published in peer-reviewed journals. Reference lists of eligible studies and review studies were also hand-searched. We included studies published from 2004—as this was the advent of widespread social media use—to May 2024 [32].

### Inclusion and Exclusion Criteria

The inclusion and exclusion criteria listed in Table 1 were applied.

**Table 1:** Inclusion and exclusion criteria for screening.

Key concepts	Inclusion criteria	Exclusion criteria
Population	Studies where the campaign targeted the general population or demographic groups, for example, men or young people. High-income countries where use and access to social media and resources for public health campaigns are comparable.	Studies where campaigns were targeted only at clinical populations or professionals. Low- and middle-income countries.
Intervention	Social media public mental health campaigns where the aim of the campaign was to raise awareness of mental health, change attitudes toward mental health, reduce stigma, and encourage help-seeking. This includes mass media campaigns that use other media, but only if there are data on the impact of the social media element.	Social media campaigns for related outcomes, such as physical activity and bullying. Campaigns solely focused on preventing suicide, serious mental illness, or the mental health impacts of COVID-19. Digital support groups. Experimental vignettes of campaigns comparing different kinds of messaging.
Outcomes	Key performance indicators and metrics related to social media use in health promotion, as defined by Neiger et al [26], which include exposure, reach, and low, medium, and high engagement. We adapted this framework to capture more outcomes related to behavior change, so high engagement outcomes also included knowledge, attitudes and beliefs, stigma, access to resources, and help-seeking intentions and behaviors.	Outcomes unrelated to exposure, reach, and engagement.
Study types	Experimental studies, quasi-experimental studies, pre- and poststudies, and cross-sectional and observational studies.	Reviews and qualitative designs.
Publication types	Peer-reviewed studies. Reports from government agencies or charities that have evaluated their social media mental health campaigns.	Theses, protocols, dissertations, conference papers, editorial letters, notes, books, comments, and meeting abstracts.
Language	Studies in English.	Studies not in English.

### Screening

Rayyan, a web-based tool, was used to screen abstracts according to the inclusion and exclusion criteria outlined in Table 1. RP reviewed all abstracts, and APK and JMS reviewed a proportion (1026/3396, 30%) of the abstracts after duplicates were removed. RP reviewed all full-text studies, and APK and JMS reviewed a proportion of full-text studies (11/69, 16%). Disagreements were resolved in discussions between reviewers and the other authors.

### Data Extraction

We extracted data using Excel (Microsoft Corp) to collect key information on study characteristics, details of the campaigns, methodological approaches, outcome measures, and key findings. To measure the success of social media campaigns, we captured outcomes derived from a version of key performance indicators and metrics related to social media use in health promotion adapted from Neiger et al [26], which includes, exposure, reach, and low-, medium-, and high-engagement (Table 2). Outcome measures capturing changes in knowledge about mental health conditions, attitudes toward mental health, stigma, and intention to seek help for mental health problems were not accounted for using the original framework, but were categorized as high engagement with a campaign, as greater knowledge and intentions are precursors to behavior change and taking actions offline [25,26,33]. We assessed changes in outcomes both before and after the campaign, as well as between individuals who were aware of the campaign and those who were not. We only reported differences that were statistically tested and for which *P* values, CIs, or effect sizes were available.

**Table 2:** Descriptions and examples of social media–related outcomes to be extracted from studies.

Outcomes	Descriptions	Examples of measures
Exposure^a^	Views of social media content	Campaign awareness, number of views (engagement with content, eg, watching videos), impressions (number of times digital content is displayed regardless of whether it is clicked or not)
Reach	Interaction with social media content and users’ characteristics	Followers, demographics of users
Low-level engagement	Agreement with the social media content	Number of likes for posts
Medium-level engagement	Users creating or sharing their own social media messages or sharing campaign messages on their own profiles	Number of posts or retweets
High-level engagement	Users’ understanding of the messaging, intention to change their behavior, or actions taken on social media or offline related to the desired behavior change	Mental health knowledge and literacy, attitudes or beliefs about mental health stigma, for example, desire for social distance, behavior change: seeking help for mental health, activities for positive mental health, and help-seeking intentions

^a^Exposure, reach, and low-, medium-, and high-level engagement were measured using a version of key performance indicators and metrics related to social media use in health promotion adapted from Neiger et al [26].

### Data Synthesis

We conducted a narrative synthesis of study findings summarizing the characteristics of social media campaigns (aims, target audience, target location, and campaign developer, eg, charity and government) [34]. The study characteristics, such as settings, design, participants, and main findings, were summarized. We organized findings by outcome by summarizing people’s exposure to the campaign (eg, campaign awareness and views and impressions) and low-medium level engagement with the campaign on social media (eg, likes and shares on social media). To summarize the campaign’s reach, especially among underserved communities, we gathered data on the demographics of the individuals reached by the campaign, where available. We summarized how effective social media campaigns were at improving high engagement, that is, people’s knowledge, attitudes, stigma (which was largely measured by the desire for social distance), and behavior change (which included intentions to seek help for mental health, service use, and activities to enhance positive mental health).

## Results

### Overview

**Figure 1:**
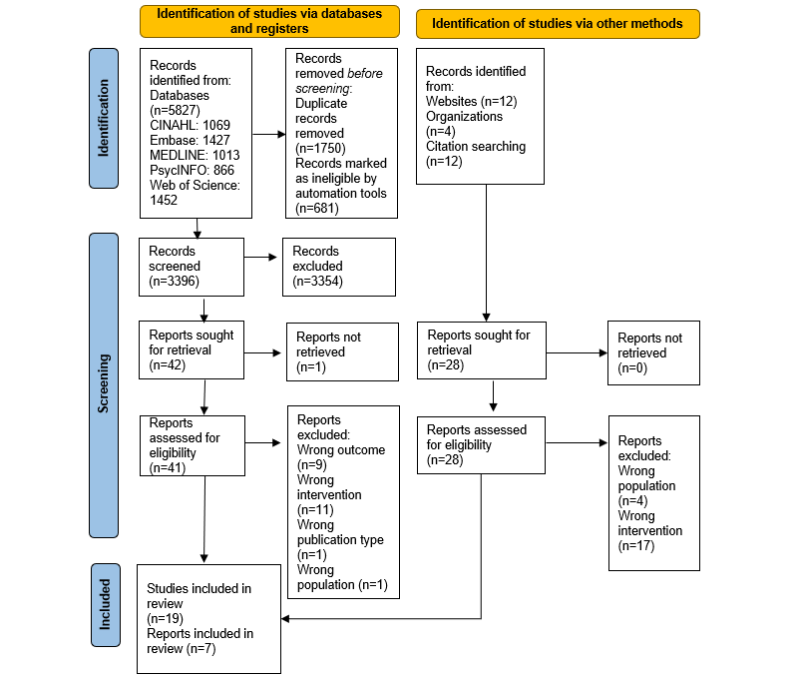
PRISMA-ScR flow diagram showing the process of inclusion and exclusion of sources. PRISMA-ScR: Preferred Reporting Items for Systematic Reviews and Meta-Analyses extension for Scoping Reviews.

A total of 5827 publications were identified from the database search, and a further 28 publications were identified from gray literature. After screening, 26 sources were included for data extraction and analysis (see PRISMA-ScR flow diagram in Figure 1).

### Study Characteristics

Table 3 describes the characteristics of the campaigns. The campaigns included in this review were mostly undertaken in the United States (10/26, 39%), followed by the United Kingdom (8/26, 31%) and Canada (4/26, 15%). The most common social media platforms used by campaigns were Facebook (13/26, 50%), followed by X (formerly Twitter; 7/26, 27%), Instagram (4/26, 15%), and YouTube (4/26, 15%), respectively. All the campaigns that reported the social media platforms they used reported delivering content on several platforms (15/26, 58%), with most campaigns using three platforms (10/26, 39%). Most of the campaigns were developed by government agencies like local or national health or public health departments (12/26, 46%) and by charities (12/26, 46%), followed by health services (10/26, 39%), universities (4/26, 15%), and sports teams (4/26, 15%). Just over half of the campaigns were developed by multiple agencies (14/26, 54%).

There were 5 campaigns that were evaluated by multiple studies: Time to Change, WhyWeRise, Act Belong Commit related campaigns, In One Voice and Action Minded, and WhatMakesUs related campaigns (see Table 4 for a summary of the social media activities of these campaigns).

**Table 3:** Study characteristics of included studies sorted by campaign and year.

Author (year)			Campaign															Publication type
			Name		Evaluation period		Aims		Target users		Target location		Developer		Social media platforms used			
**Time to change campaigns**																		
	Evans-Lacko et al (2013) [35]	Time to Change (phase 1)		2009-2011		To reduce stigma and discrimination		Mid-20s to mid-40s, from middle-income groups		United Kingdom		Charity		Facebook, Twitter, and YouTube		Journal study		
	Henderson et al (2016) [36]	Time to Change (phases 1-2)		2008-2015		To reduce stigma and discrimination		Mid-20s to mid-40s, from middle-income groups		United Kingdom		Charity		NR^a^		Journal study		
	Henderson et al (2017) [37]	Time to Change (phase 2)		2012-2016		To reduce stigma and discrimination To increase help-seeking		Mid-20s to mid-40s, from middle-income groups		United Kingdom		Charity		NR		Journal study		
	Sampogna et al (2017) [38]	Time to change (phases 1-2)		2009-2014		To reduce stigma and discrimination		Mid-20s to mid-40s, from middle-income groups		United Kingdom		Charity		Twitter and Facebook		Journal study		
	González-Sanguino et al (2019) [39]	Time to Change (phase 3)		2017-2019		To reduce stigma and discrimination To increase help-seeking To address inequalities in demographic groups		Mid-20s to mid-40s, middle- to low-income groups, men, parents		United Kingdom		Charity		Facebook, Twitter, Instagram, and Snapchat		Journal study		
	Henderson et al (2020) [40]	Time to Change (phases 1-3)		2008-2019		To reduce stigma and discrimination To increase help-seeking To address inequalities in demographic groups		Mid-20s to mid-40s, middle- to low-income groups, men, parents		United Kingdom		Charity		NR		Journal study		
**WhyWeRise campaign**																		
	Collins et al (2018) [41]	WhyWeRise		2018		To reduce stigma To increase awareness of how to seek mental health care.		Ages 14-24 years		Los Angeles, California, United States		Multiple—government bodies and health services		Facebook, Instagram, and Twitter		Report		
	Collins et al (2020) [42]	WhyWeRise		2019		To reduce stigma To increase awareness of how to seek mental health care		Ages 14-24 years		Los Angeles, California, United States		Multiple—government bodies and health services		Facebook, Instagram, and Twitter		Report		
	Collins et al (2022) [43]	WhyWeRise		2021		To create awareness of two key resources and drive residents to them: the website and the Helpline		Ages 14-24 and Hispanic, Black, and Asian residents		Los Angeles, California, United States		Multiple—government bodies and health services		Facebook, Instagram, and Twitter		Report		
	Collins et al (2022) [44]	WhyWe Rise—L.A. Dodgers Mental Health Campaign		2020-2021		To raise awareness of resources and stigma-reduction		Hispanic residents		Los Angeles, California, United States		Multiple—sports teams, government bodies, and health services		Facebook, Instagram, and Twitter		Report		
	Collins et al (2022) [45]	WhyWe Rise—L.A. Dodgers Mental Health Campaign		2022		To raise awareness of resources and stigma-reduction		Hispanic residents		Los Angeles, California, United States		Multiple—sports teams, government bodies, and health services		Facebook, Instagram, and Twitter		Report		
**ABC^b^ and related campaigns**																		
	Drane et al (2022) [46]	Act-Belong-Commit		2018-2019		To improve mental health literacy and reduce stigma To stimulate people to undertake activities conducive to good mental health		General public		Western Australia, Australia		Multiple—universities and government bodies		NR		Journal study		
	Santini et al (2022) [47]	The ABCs of Mental Health		2019-2021		To encourage individuals to engage in mentally healthy behaviors		General public		Denmark		University		Facebook, Instagram, and LinkedIn		Journal study		
**In One Voice campaign**																		
	Livingston et al (2013) [48]	In One Voice		2012		To raise mental health awareness and improve attitudes toward mental health issues		Ages 13-25 years		British Columbia, Canada		Multiple—sports teams, government bodies, and health services		Facebook, Twitter, and YouTube		Journal study		
	Livingston et al (2014) [49]	In One Voice		2012-2013		To raise mental health awareness and improve attitudes toward mental health issues		Ages 13-25 years		British Columbia, Canada		Multiple—Sports team, government body, and health services		Facebook, Twitter, and YouTube		Journal study		
**WhatMakesUs and Action Minded campaigns**																		
	Public Goods Project (2019) [50]	Action minded		2018-2019		Reducing mental health stigma		General public		Several States, United States		Charity		NR		Report		
	Diouf et al (2022) [51]	WhatMakesUs and Spokesimals Midwest		2020-2021		Reducing mental health stigma		General Public		Several Midwestern States, United States		Multiple- charity and wellness consultancy		NR		Journal study		
	Alvarado-Torres et al (2023) [52]	WhatMakesUs		2021-2022		Reducing mental health stigma		General Public		Several Midwestern States, United States		Multiple- charity and wellness consultancy		Facebook and Instagram		Journal study		
**Other campaigns**																		
	Hann and Hemming (2016) [53]	Six Ways to Wellbeing Campaign		2014-2016		To encourage people to improve their mental well-being		General public		Kent, United Kingdom		Multiple—government bodies and health services		Twitter, Facebook, and YouTube		Report		
	Hahn et al (2023) [54]	Every Mind Matters		2019-2022		To encourage people to improve their mental well-being		General public		United Kingdom		Government body		NR		Journal study		
	Hansson et al (2016) [55]	Hjarnkoll		2009-2014		To reduce stigma		8 regions in Sweden and some national reach		Sweden		Government body		NR		Journal study		
	Booth et al (2018) [24]	Bell Let’s Talk		2006-2015		To reduce stigma		General public		Ontario, Canada		Telecommunications company		NR		Journal study		
	Zenone et al (2020) [56]	Everything Is Fine		Not reported		To raise awareness of resources		Ages 12-17, men		British Columbia, Canada		Multiple—charity and health services		Instagram, Snapchat		Journal study		
	Collins et al (2019) [57]	Each Mind Matters		2014-2016		To improve mental health To increase help-seeking		General public		California, United States		Government body		NR		Journal study		
	Thompson et al (2021) [58]	Look Around		2017-2018		To reduce stigma and increase help-seeking		Ages 11-18 in one Midwestern County		Midwestern county, United States		Multiple—schools, universities, health services, and students		NR		Journal study		
	Coughlan et al (2021) [59]	#YMHanimate		2019-2020		To develop engaging public mental health animations		Ages 16-25		Ireland		Multiple—universities and charities		Twitter, Facebook, and YouTube		Journal study		

^a^NR: Not reported.

^b^ABC: Act-Belong-Commit.

**Table 4:** Summary of the social media activities for the most common campaigns.

Campaign	Key social media components
Time to Change	Used social media to deliver key messages and encourage behavior change through small actions, such as starting conversations about mental health with friends. It suggested simple ways to change behavior and recruited individuals to engage in local campaign activities.
WhyWeRise	Partnered with the Los Angeles Dodgers (MLB^a^ team) to expand reach, especially to Hispanic residents. It promoted community engagement with mental health issues and created a movement to address barriers to mental health access using interviews and stories.
Act Belong Commit	Encouraged people to be physically, spiritually, socially, and mentally active in ways that increase their sense of belonging to their communities.
In One Voice	Launched by the Vancouver Canucks (hockey team) in collaboration with local health authorities and charities. It included a 2-minute public video featuring a popular Canucks player discussing mental health issues and promoting the Mindcheck website. They encouraged viewers to pledge support for friends and family members with mental health issues by creating and submitting videos to the Mindcheck website. It featured in several venues and forums, including a home game of the Vancouver Canucks.
Action Minded or WhatMakesUs	Contact-based campaign inviting individuals with mental health conditions to share video and photo testimonials of their personal experiences with mental health and stigma. Community-based organizations received tailored images and videos each month, focusing on new themes related to mental health stigma.

^a^MLB: Major League Baseball.

### Key Findings of Campaign Evaluations by Outcome

#### Exposure

##### Campaign Awareness

Multimedia Appendix 3 [24,35-59] provides a table of the key findings of the studies. Campaign awareness was measured using surveys after campaigns by almost three-quarters (19/26, 73%) of the studies. Only 5 of 19 (26%) studies including this measure reported campaign awareness above 50% at any given time [38,42,44,46,52]. The highest level of campaign awareness was during the 2012 promotion of the UK Time to Change campaign, with 81.7% of respondents reporting campaign awareness [38]. The lowest level was 12%, reflecting poorer campaign awareness for The ABCs of Mental Health campaign in Denmark [47].

Of the studies assessing campaign awareness, around a third (7/19, 37%) measured this outcome at several points throughout the duration of the campaigns. For the Time to Change campaign*,* 2 studies found that there was an increase in campaign awareness in phase one of the campaign, rising from 39% in 2009 to 81.7% in 2012 [35,38]. During phase two, 2 studies found awareness decreased between 2013 and 2014 to around 20% in 2014 [37,40], and 1 study found it remained the same in phase three (~33%) from 2017 to 2018 [39]. The US WhyWeRise campaign showed an initial increase in campaign awareness, rising from 20% in 2018 to 50% in 2020, but this dropped to 37% in 2021 [41,44]. The Canadian In One Voice campaign showed an increase in campaign awareness, from 25% in 2012 to 49% in 2013 [49]. The US WhatMakesUs campaign awareness increased from 30% in 2020 [51] to 53% during 2020-2022 [52].

##### Views and Impressions

Over a third of the studies (9/26, 35%) reported social media campaign exposure outcomes by recording digital metrics such as views and impressions [38,41,48,49,52,53,56,59,60] (Multimedia Appendix 3 [24,35-59]). A total of 3 of 9 (33%) studies found that Facebook was the social media platform that accumulated the most views, compared to other platforms; all these campaigns targeted adults [41,52,59]. In the campaign in 2019-2020, Facebook views were found to account for nearly two-thirds of the campaign’s social media views, making up 10,437 of the total 15,848 views [59]. In the 2021-2022 campaign, out of a total of 2,558,291 impressions, Facebook accounted for 1,838,300 impressions whilst Instagram accounted for 719,991 impressions [52].

#### Reach

Under half of the studies (11/26, 42%) reported measures of reach (who their campaigns targeted) [35,39-41,43-45,50,52,53,59] (Multimedia Appendix 3 [24,35-59]). Table 5 provides a summary of the reach of the campaigns by key demographic characteristics. Campaign awareness varied significantly across different demographic factors. In some campaigns targeting ethnic minority groups, there was higher initial exposure among Black and Hispanic groups, but lower awareness among Asian respondents. Women were generally more aware of the campaigns than men, and younger age groups showed higher awareness. Socioeconomic status had mixed results. Additionally, factors such as having children, familiarity with mental illness, living in specific regions, and lower education levels were associated with increased campaign awareness. Campaigns that aimed to target underserved populations appeared to do so effectively by using different languages and working with popular sports teams and mental health activists to share and create information [44,45,50].

**Table 5:** Summary of the reach of the included social media campaigns.

Demographic factor	Studies reporting reach (n=26), n (%)	Key findings
Ethnicity	7 (27)	Time to change: increased exposure for those of Black ethnicity initially, but this declined in the latest evaluation [35,39,40]. WhyWeRise: increased exposure for Hispanic populations but lower awareness among Asian respondents [43-45]. WhatMakesUs: increased exposure for non-Hispanic White respondents [52].
Gender	7 (27)	Most studies found increased campaign awareness among women compared to men (4/7, 57%) [35,52,53,59]. A total of 2 of 7 (29%) studies found that men were more likely to be aware of campaigns than women [39,45].
Age	5 (19)	Younger age groups were more likely to be aware of campaigns. [41,43-45,52,59]
Socioeconomic group	2 (8)	Mixed findings: lower awareness among lower socioeconomic groups in one study [40]; greater awareness among lower-income individuals in another [43].
Other demographics	3 (12)	Factors such as having children, familiarity with mental illness, ever having a mental health condition, living in London or the East Midlands regions of the United Kingdom, and having a high school degree or less were associated with campaign awareness [39,43,50].

#### Low-Medium Engagement With Campaigns

Low engagement, such as the number of likes a campaign post received, was reported in 4 of 26 (15%) studies [48,50,52,53]. Low engagement varied widely across campaigns, with one campaign receiving 758 likes on Facebook [53], whereas another reported 23,763 Facebook engagements [52]. In two campaigns targeted at adults, one in 2014-2016 and one in 2021-2022, Facebook received more engagements than Instagram, Twitter, and YouTube [52,53]. Medium engagement, such as sharing and reporting campaign content, was reported in 2 of 26 (8%) studies; one reported 78,520,289 retweets [24] and one only 2563 [53]. Large quantities of views did not necessarily equate to large quantities of active engagement with the campaign. For example, the US WhatMakesUs campaign had a total of 2,558,291 impressions, but only 27,053 engagements (likes, comments, shares, or post clicks), and 92,313 website visits [52]. In a local UK campaign, there were 246,255 X (formerly Twitter) impressions and 440 Tweet likes during the campaign.

#### High-Level Engagements

##### Overview

Most of the studies (23/26, 89%) reported indicators of high engagement (Multimedia Appendix 3 [24,35-59]). Studies used cross-sectional surveys to assess changes in these outcomes. Over half of the studies (14/23, 61%) compared these outcomes before and after the campaign. Almost three-quarters (17/23, 74%) compared these outcomes between those who were aware of the campaign and those who were not. Attitudes about mental health (17/23, 74%) and stigma (17/23, 74%) were most frequently reported, followed by mental health knowledge (16/23, 70%) and behavior change (15/23, 65%). Multimedia Appendix 4 shows a breakdown of high-engagement outcomes by the 5 main campaigns that were evaluated across multiple studies, and the sections below provide a more detailed breakdown of these outcomes.

Across all studies, stigma, as measured by the desire for social distance (5/11, 45%), and attitudes about mental health (5/11, 45%) most frequently showed improvement after campaigns (Multimedia Appendix 5). Just over a third of studies (4/11, 36%) found that knowledge about mental health improved after campaigns. Behavior change showed the least positive change over time, with only 1 of 8 (13%) studies reporting a significant improvement in seeking help for mental health postcampaign compared to precampaign. Many studies across these outcomes found mixed findings, in which changes occurred for some items of an outcome measure but not others, or when changes were evident in one demographic group but not in others.

Conversely, analyses of postcampaign surveys comparing outcomes between individuals who were aware of the campaigns and those who were not found that increased awareness was most often associated with positive behavior changes (including seeking help for mental health, activities for positive mental health, and help-seeking intentions; 12/12, 100%). However, increased awareness was least frequently associated with positive changes in attitudes toward mental health (3/11, 27%). Over half of the studies showed that increased awareness was associated with reduced stigma (7/12, 58%) and improved mental health knowledge (6/11, 55%) (Multimedia Appendix 6).

##### Knowledge

The most common tool used to measure knowledge was the Mental Health Knowledge Schedule, which was used by just under half of the studies measuring knowledge (7/16, 44%) [35,36,38-40,55,60]. In total, 1 of 16 (6%) studies in this group used the Mental Health Literacy—Recognition scale and the Mental Health Literacy—Action scale [54]. Other studies used their own scales, with some adapted from the Mental Health Knowledge Schedule.

Most of the studies assessing knowledge improvement before and after the campaign, most (5/11, 45%) found no significant change [35,38,39,48,49], and less than a third (4/11, 36%) found an improvement post campaign [36,40,50,55]. A total of 2 of 11 (18%) studies reported mixed findings [51,61]; knowledge improved in some areas, such as for agreement that medication can be an effective treatment for people with mental health conditions, but no changes were found for agreement that therapy can be effective [51]; or knowledge improvement was not sustained [61].

In the postcampaign surveys, most studies (6/11, 55%) that assessed knowledge differences between those aware of the campaign and those unaware found that campaign awareness was associated with greater knowledge [35,38,39,47,50,61]. A total of 4 of 11 (36%) studies found no significant difference [41,42,51,57], and 1 of 11 (9%) studies reported mixed findings; knowledge related to the effectiveness of therapy and counseling, but not medication, improved in the campaign-aware group [52].

##### Attitudes

The Community Attitudes to Mental Illness was the most widely used tool for capturing attitudes and was used in 7 of 17 (41%) studies [35,36,38-40,55,60]. In total, 2 of 17 (11%) studies used a scale developed by another author [48,49]. Other studies used their own scales, with some adapted from the Community Attitudes to Mental Illness.

Of the studies comparing change in attitudes before and after the campaign, many (5/11, 45%) showed improvement post campaign [36,40,49,50,55]. The same proportion of studies (5/11, 45%) reported no significant change in attitudes post campaign [35,38,39,48,51], and 1 of 11 (9%) studies reported mixed changes whereby there was significantly less change in African American respondents compared to White respondents post campaign [58].

Only 3 of 11 (27%) studies found more positive attitudes in campaign-aware individuals compared to noncampaign-aware individuals [38,50,51]. A total of 3 of 11 (27%) studies found no significant difference [39,41,52], 5 of 11 (45%) studies reported mixed findings, in which campaign awareness was associated with some attitude measurement items but not others [35,42,43,45,57]. For example, 1 study found no significant change for the item about whether people with mental health problems should not be given any responsibility [35]. In the WhyWeRise campaigns in 2019, they found that campaign-aware individuals, compared to noncampaign-aware individuals, tended to agree with one of the negative stereotypes they assessed, that those who have had a mental illness will never contribute much to society [42]. In 2021, they found that campaign-aware individuals compared to noncampaign-aware individuals tended to agree with two negative stereotypes, that a person with a mental illness is a danger to society and that people who have had a mental illness are never going to be able to contribute much to society [43]. In the latest evaluation, in 2022, it was found that campaign-aware young people, compared to those who were unaware, were significantly more likely to report a desire to delay seeking mental health treatment out of fear of others finding out, but no other attitudes differed and this did not differ for adults [45].

##### Stigma

Just over half of the studies (9/17, 53%) used their own scales measuring stigma via measuring desire for social distance, with most adapted from the Reported and Intended Behavior Scale. The Reported and Intended Behavior Scale was used in just under half of the studies (8/17, 47%) assessing stigma.

Of the studies comparing stigma before and after the campaign, just under half (5/11, 45%) reported a reduced desire for social distance post campaign [36,40,49,51,55]. In total, 3 of 11 (27%) studies reported no significant change in desire for social distance from baseline to follow-up [38,39,50], and 2 of 11 (18%) studies showed mixed findings [35,48]. In the UK Time to Change campaigns, Evans-Lacko et al [35] found only a single item about being willing to live with someone with a mental health problem showing significant improvement post campaign [48]. In the Canadian In One Voice campaign, only one item about willingness to invite someone with a mental illness to their home significantly improved post campaign [48]. Only 1 of 11 (9%) studies found desire for social distance increased slightly after the campaign in the United Kingdom [61].

Of the studies assessing desire for social distance in “campaign aware” individuals compared to “noncampaign aware” individuals, over half (7/12, 58%) found a reduced desire for social distance in those who were campaign aware [35,38,39,44,50,51,61]. In total, 2 of 12 (17%) studies found no significant change [42,43], and 3 of 12 (25%) studies reported mixed findings [41,45,52]. For example, Alvarado-Torres et al [52] found that actual behaviors related to social distancing, such as living with a person with a mental health condition, showed significant improvement, whereas intended behaviors, such as willingness to live with a person with a mental health condition, showed nonsignificant improvement. In the earliest evaluation of the US WhyWeRise campaigns in 2018, campaign awareness was associated with a willingness to work closely with someone who has a serious mental illness, but not moving next door to or socializing with such a person [41]. In the latest evaluation in 2022, campaign awareness was associated with less desire for social distancing in adults but was not for young people [45].

##### Behavior Change

Studies most commonly measured self-reported help-seeking intentions (6/12, 50%) [37,46,48,49,58,61] or help-seeking actions, including service, website, and helpline use that was mostly self-reported (5/12, 42%) [24,43-45,57]. A third of the studies (4/12, 33%) measured activities to enhance mental health [47,50-52].

Of the studies measuring behavior change before and after the campaign, Booth et al [24] (1/8, 13%) showed a positive change at follow-up in service use. A total of 2 of 8 (25%) studies described mixed findings [49,58]. Livingston et al [49] found that the measurement item about “making an effort to learn about accessing mental health services” was the only one to show significant improvement postcampaign. Thompson et al [58] found that an increase in help-seeking intentions improved overall, but African American respondents reported less improvement post campaign than White respondents. A total of 3 of 8 (38%) studies found no significant change in behavior (activities to enhance mental health) post campaign [48,50,51], and 2 studies observed a decline post campaign in help-seeking intentions, including intentions to visit a general practitioner for mental health concerns and self-rated help-seeking and psychological well-being self-efficacy [37,61]. All the studies (12/12, 100%) measuring behavior change by campaign awareness found a positive association.

## Discussion

### Principal Results

Most studies evaluating social media-based mental health campaigns have used serial cross-sectional surveys to assess campaign effectiveness by measuring exposure, reach, and engagement. Campaign awareness varied across campaigns and by demographic factors, including ethnicity, gender, age, and socioeconomic group. Younger people and women were most consistently reached by public mental health campaigns. Most studies measured changes in knowledge, stigma, and attitudes, and over half of all studies reported on behavior change outcomes such as help-seeking. Stigma and attitudes toward mental health were found to improve most post campaign compared to precampaign, and behavior change outcomes showed the least improvement. However, post campaign, those who were aware of campaigns were much more likely than those who were unaware to change their behaviors, such as seeking help for mental health concerns, engaging in activities for positive mental health, and intending to seek help.

This review showed that mental health knowledge, stigma, and attitudes generally improved before and after campaigns. These findings could reflect general societal shifts in these outcomes over the same period rather than be directly attributable to the campaigns. For example, many high-income countries have seen a general trend of reduced mental health stigma over the past 30 years, especially concerning common mental health problems like depression [62,63]. However, some studies found that campaign awareness was associated with negative attitudes about mental health. These included believing that those who have had a mental illness will never contribute much to society, that a person with a mental illness is a danger to society, and a desire to delay seeking mental health treatment out of fear of others finding out [42,43,45]. These findings came from the US WhyWeRise campaign, and it was suggested that the reasons for these attitudes could be due to the campaign inadvertently focusing more attention on some of the challenges that can be associated with mental health problems rather than empowerment [45]. Previous research also indicates that improving knowledge and awareness might not have long-lasting changes in attitudes, stigma, and behavior change [48,64]. Furthermore, analysis of social and traditional media over the past 20 years has shown that the media generally has failed to move away from negative stereotypes, such as associating some mental health disorders with danger and fear [65,66]. Although these campaigns likely aimed to challenge these perceptions, they seem to have not always succeeded in combating the stereotypes perpetuated by other media sources. Research suggests that future public mental health campaigns should move away from promoting a biomedical explanation of mental health problems that can perpetuate the othering of individuals affected by mental distress. Instead, campaigns should consider the public understanding of mental health and frame mental health problems as a response to social, economic, political, and biological factors [64,67]. Research on identifying effective and ineffective content and messaging strategies is crucial for framing campaigns appropriately and avoiding the perpetuation of negative stereotypes [68].

All evaluations that measured behavior change showed that those who were campaign aware were more likely to change their health behaviors after the campaigns than those who were unaware of the campaigns. These behaviors included self-reported intentions to seek help and engage in activities to enhance mental health, but also objective measures, such as an increase in the use of mental health outpatient services, helplines, and websites. However, we cannot be certain the behavior changes were due to the campaigns, as they did not account for all confounding factors. For example, the campaign-aware group might have been more likely to seek help because they were experiencing more distress than those who were unaware of the campaigns. Additionally, when behavior change was measured before and after the campaigns, few evaluations found any change in behaviors. In some evaluations of UK campaigns, help-seeking intentions decreased, possibly due to increased difficulty accessing mental health care in many parts of the United Kingdom [37,61,69]. This suggests that being aware of campaigns may be key to achieving behavior change. However, these behavior changes tend not to be sustained and need to be supported by improved access to services. Individual behavior change alone is insufficient; changes in services are also necessary for a sustained impact on mental health. Fragmented systems and long waiting lists pose a barrier to behavior change, as they can inhibit opportunities, as suggested by the Capability, Opportunity, Motivation, and Behavior Change model [25]. Public health campaigns alone cannot address these issues.

We found that campaign awareness across these social media campaigns was more common for younger age groups and women. This is likely partly because most social media users are younger and, therefore, more exposed to the campaigns. However, evidence suggests that globally, men are more likely than women to use platforms like Facebook, which were used by many of the campaigns in this review [70]. It may be that more women are campaign aware because data show that this group has experienced the sharpest increases in mental health problems over the last 20 years in many high-income countries, so these campaigns are of more relevance to them, and they are more aware [71-73]. However, there is a real need to reach young men, as they have significantly higher suicide rates compared to women [74]. Some studies also showed success at targeting populations who are unequally affected by mental health or have poor access to services, such as ethnic minorities and those in lower socioeconomic groups. However, the reach of campaigns was not reported in most studies, so it is unclear if they are reaching those who might benefit most and the extent to which they can address inequalities in mental health access and support. Campaigns that seemed to successfully target these populations were those that used different languages, popular sports teams, and mental health activists to share and create information [44,45,50]. In total, 1 study found that there was less improvement in changes in attitudes and behaviors post campaign in African American respondents compared to White respondents in the United States, suggesting that some campaigns may have less positive impact on underserved groups. Future campaigns should leverage the benefits of social media to reach different underserved groups using content from influencers and activists popular with the target groups.

### Limitations

This was a scoping review, so we have not assessed included studies for quality. However, this design was necessary to scope out what is known and what has been done in a short time period across a wide range of literature, to understand the effectiveness of social media–based public mental health campaigns. As we have only included studies written in English and focused on high-income settings, the findings of this review may not be generalizable to non-English-speaking countries. We chose only to include studies from high-income countries to more easily compare campaigns across countries with similar public health resources, so these findings may not be relevant to low-middle-income countries. The field of social media research is expanding rapidly, and this scoping review only captures a snapshot of evidence related to social media campaigns across platforms that were most popular during the period of 2008-2022. More recent campaigns will likely adapt their methods and messaging to newer platforms such as TikTok, and this may prove to have a different impact on outcomes. All studies used cross-sectional surveys and had multiple campaign components, so it is not possible to know whether changes that occurred in the outcomes were caused by the social media campaigns or some other factor that changed over time. The studies that compared those who were campaign aware and not aware had issues with confounding variables, making it difficult to attribute changes solely to campaign awareness. Studies also assessed outcomes over varying lengths of time, and some used nonstandardized measures of knowledge, attitudes, and stigma, which makes it difficult to compare outcomes across campaigns. Many studies used self-report measures to evaluate behavior change, such as their intention to visit a mental health practitioner, so it may not reflect actual changes in behavior. The review was limited because it excluded qualitative studies that would have provided deeper insights into how the campaign messaging is understood, which aspects are most impactful, and why some outcomes may be negative.

### Conclusions

This scoping review underscores the potential of social media campaigns in improving knowledge, attitudes toward mental health, and stigma, and promoting help-seeking behavior. It highlights the potential importance of campaign awareness in contributing to behavior change and the need for more targeted campaigns to reach underserved communities, who may benefit from better mental health information, access to resources, and a focus on empowerment. However, we still have questions regarding whether these social media campaigns achieve more than traditional media campaigns and if we are fully capitalizing on their potential for extended reach. Future research should more consistently measure reach and behavior change outcomes to understand which groups benefit from these campaigns and how these campaigns affect help-seeking behaviors. Future campaigns need to consider how the public understands mental health, how to present information to not perpetuate mental health stigma, and how campaigns could be designed to achieve sustained benefits. Furthermore, as the field of social media research continues to evolve rapidly, ongoing evaluation of new platforms and campaign strategies will be crucial to maximize the impact of these interventions on public mental health.

## Appendix

Multimedia Appendix 1 PRISMA-ScR checklist.

Multimedia Appendix 2 Search Strategy for MEDLINE.

Multimedia Appendix 3 Table of the key findings of campaign evaluations.

Multimedia Appendix 4 Summary of outcomes by campaigns.

Multimedia Appendix 5 Percentage of articles showing changes in knowledge, attitudes, stigma and behavior change before and after the social media campaigns.

Multimedia Appendix 6 Percentage of articles presenting analyses showing the relationship between social media campaign awareness and knowledge, attitudes, stigma and behavior change.

## Abbreviations

COM-B: Capability, Opportunity, Motivation, and Behavior Change
LGBTQ+: lesbian, gay, bisexual, transgender, queer/questioning, and others
PRISMA-ScR: Preferred Reporting Items for Systematic Reviews and Meta-Analyses extension for Scoping Reviews
