# Supplementary material for: The Effectiveness of Social Media Campaigns in Improving Knowledge and Attitudes Toward Mental Health and Help-Seeking in High-Income Countries: Scoping Review
Source: J Med Internet Res. 2025 May 23;27:e68124. doi: 10.2196/68124 (PMC12144482; doi:10.2196/68124)
Supplement: Multimedia Appendix 2 [file jmir_v27i1e68124_app2.docx]

| 1. | Social Media/ |
| --- | --- |
| 2. | Online Social Networking/ |
| 3. | (social adj2 (networking or media)).kf,tw. |
| 4. | facebook.kf,tw. |
| 5. | twitter.kf,tw. |
| 6. | instagram.kf,tw. |
| 7. | snapchat.kf,tw. |
| 8. | youtube.kf,tw. |
| 9. | Reddit.kf,tw. |
| 10. | TikTok.kf,tw. |
| 11. | Whatsapp.kf,tw. |
| 12. | Social Marketing/ |
| 13. | social marketing.kf,tw. |
| 14. | Mental Health/ |
| 15. | (Mental adj well*).kf,tw. |
| 16. | (Mental adj2 health*).kf,tw. |
| 17. | (wellbeing or well-being).kf,tw. |
| 18. | Health Knowledge, Attitudes, Practice/ |
| 19. | Help Seeking Behavior/ |
| 20. | awareness.kf,tw. |
| 21. | social stigma/ |
| 22. | stigma*.kf,tw. |
| 23. | attitude*.kf,tw. |
| 24. | (help-seeking or help seeking).kf,tw. |
| 25. | 1 or 2 or 3 or 4 or 5 or 6 or 7 or 8 or 9 or 10 or 11 or 12 or 13 |
| 26. | 14 or 15 or 16 or 17 |
| 27. | 18 or 19 or 20 or 21 or 22 or 23 or 24 |
| 28. | 25 and 26 and 27 |
| 29. | limit 42 to (english language and yr="2004 -Current") |
