# Supplementary material for: The Effectiveness of Social Media Campaigns in Improving Knowledge and Attitudes Toward Mental Health and Help-Seeking in High-Income Countries: Scoping Review
Source: J Med Internet Res. 2025 May 23;27:e68124. doi: 10.2196/68124 (PMC12144482; doi:10.2196/68124)
Supplement: Multimedia Appendix 3 [file jmir_v27i1e68124_app3.docx]

**Multimedia Appendix 2: Table of the key findings of campaign evaluations**

| **First Author, Year** | **Study design, Sample, Campaign year(s) evaluated** | **Outcomes (survey measure) ^[[1]](#footnote-2)^** | **Key findings^[[2]](#footnote-3)^** | | |
| --- | --- | --- | --- | --- | --- |
| **Time to change campaigns** | | | | | |
| Evans-Lacko, 2013 [35] | Surveys over multi cross-sectional waves  Ages 25-45,  middle income groups  n=900-1,100 per survey. Total n= 5,615  2009-2011 | 1. Exposure - Campaign awareness | Campaign awareness ranged from 39% of sample in 2009 to 47% in 2011. | | |
|  |  | 1. Reach | Those of Black ethnicity (vs white) and females (vs males) were more likely to be aware of campaign but those of Asian ethnicity were less (vs White ethnicity). | | |
|  |  | ***(Outcome)*** | ***Baseline vs Follow-up*** | | ***CA vs NCA^[[3]](#footnote-4)^*** |
|  |  | 1. Knowledge (MAKS)^[[4]](#footnote-5)^ | 🢬 No significant improvement in overall knowledge over the campaign. | | 🡩 Campaign awareness was a significant predictor of better knowledge. |
|  |  | 1. Attitudes (CAMI) ^[[5]](#footnote-6)^ | 🢬 Attitudes about mental health did not change over the campaign. | | ⬄ Campaign awareness predicted significantly fewer stigmatising attitudes across commonality and dangerousness items, but not the responsibility item. |
|  |  | 1. Stigma -Desire for social distance (RIBS)^[[6]](#footnote-7)^ | ⬄Only 1 item in intended behaviours scale about being willing to live with someone with a mental health problem had increased over the campaign from 29.3% to 44.4% being willing. | | 🡩 Campaign awareness was a significant predictor for overall reduced desire for social distance. |
| Henderson, 2016 [36] | Surveys over multi cross-sectional waves  Ages 16-98,  n= ~1,700 per year. Total n= 12,131  2008-2015 | ***(Outcome)*** | ***Baseline vs Follow-up*** | | |
|  |  | 1. Knowledge (MAKS) | 🡩Knowledge improved by 0.17 standard deviation units (SDU) in 2015 vs 2009 | | |
|  |  | 1. Attitudes (CAMI) | 🡩Attitudes improved 0.20 SDU in 2015 vs 2008 | | |
|  |  | 1. Stigma -Desire for social distance (RIBS) | 🡩Reduced desire for social distance by 0.17 SDU in 2015 vs 2009 | | |
| Henderson, 2017 [37] | Surveys over multi cross-sectional waves  Ages 16-98,  n= ~1,700 per year. Total n= 8,659  2012-2016 | 1. Exposure - Campaign awareness | Campaign awareness ranged from 20.2% in 2014 to 43.3% in 2013. | | |
|  |  | ***(Outcome)*** | ***Baseline vs Follow-up*** | | ***CA vs NCA*** |
|  |  | 1. Behaviour change – Help-seeking intentions | 🡫 Those in 2016 were less likely to seek help from a GP than 2012 (77.7% vs 82.7%). | | 🡩 Those who were campaign aware were more likely to seek help from GP (odds ratio (OR) = 1.18, 95% CI 1.03–1.36). |
| Sampogna, 2017 [38] | Surveys over multi cross-sectional waves  Ages 16-98,  n= ~900-1,100 per survey. Total n= 10,526  2009-2014 | 1. Exposure - Campaign awareness | Campaign awareness ranged from 39% in 2009 to 81.7% in 2012. | | |
|  |  | 1. Exposure – Views | The global number of social media time to change users has increased significantly over time (100k users in 2011 vs 250k views in 2014). More people accessed Facebook and Twitter than website. | | |
|  |  | ***(Outcome)*** | ***Baseline vs Follow-up*** | | ***CA vs NCA*** |
|  |  | 1. Knowledge (MAKS) | 🢬 No significant change over time. | | 🡩 Campaign awareness was found to be associated with higher knowledge (OR = 0.95, CI = 0.68 to 1.21; P < 0.001). |
|  |  | 1. Attitudes (CAMI) | 🢬 No significant change over time. | | 🡩 Campaign awareness was associated with ‘tolerance and support’ attitudes (OR = 0.12, CI = 0.09 to 0.16; P < 0.001). |
|  |  | 5. Stigma -Desire for social distance (RIBS) | 🢬 No significant change over time. | | 🡩 Campaign awareness was associated with reduced desire for social distance (OR = 0.71, CI = 0.51 to 0.92; P < 0.001). |
| Gonzalez, 2019 [39] | Surveys over multi cross-sectional waves  Ages 25-45.  Total n= 3,700  2017-2019 | 1. Exposure - Campaign awareness | Campaign awareness ranged from 33% in 2017 and 34% in 2018. | | |
|  |  | 1. Reach | Factors associated with campaign awareness for one or more bursts included having children, familiarity with mental illness, male sex, being Black, Asian or other ethnic minorities and living in London or the East Midlands regions. | | |
|  |  | ***(Outcome)*** | ***Baseline vs Follow-up*** | | ***CA vs NCA*** |
|  |  | 1. Knowledge (MAKS | 🢬 No significant change over time. | | 🡩 Being aware of the campaign was found to be associated with higher knowledge (β = 0.60, CI = 0.36 to 0.84; p < 0.001). |
|  |  | 1. Attitudes (CAMI) | 🢬 No significant change over time. | | 🢬 Campaign awareness was not associated with improved attitudes |
|  |  | 1. Stigma -Desire for social distance (RIBS) | 🢬 No significant change over time. | | 🡩 Campaign awareness was associated with reduced desire for social distance β = 0.58, CI = 0.31 to 0.84; p < 0.001). |
| Henderson, 2020 [40] | Surveys over multi cross-sectional waves  Ages 16-98,  n= ~1,700 per survey. Total n= 19,104  2008-2019 | 1. Exposure - Campaign awareness | Campaign awareness ranged from 21.6% in 2014 and 44.4% in 2013. | | |
|  |  | 1. Reach | Differences in awareness over total campaign varied by ethnicity (White 28.1%, Black 25.1% and Asian 18.9%) and 19.2% of target socioeconomic group was aware. | | |
|  |  | ***(Outcome)*** | ***Baseline vs Follow-up*** | | |
|  |  | 1. Knowledge (MAKS | 🡩There was an increase in mental health knowledge in 9.9% of people from 2009 to 2019. | | |
|  |  | 1. Attitudes (CAMI) | 🡩12.6% of people’s attitudes improved from 2008 to 2019. | | |
|  |  | 1. Stigma -Desire for social distance (RIBS) | 🡩The level of desire for social distance decreased in 11.6% of people since 2009. | | |
| **WhyWeRise campaign** | | | | | |
| Collins, 2018 [41] | Cross-sectional Survey  Ages 14-24,  Los Angeles, USA,  n=1008  2018 | 1. Exposure - Campaign awareness | ~20% of respondents were campaign aware. | | |
|  |  | 2. Exposure – Impressions | 380,114 Instagram impressions between May 24^th^ and June 6^th^. Almost 1,500,000 Facebook impressions over a 28-day period. Over 1,400,000 Twitter (X) impressions between May 4^th^ and June 11^th^. | | |
|  |  | 3.Reach | Campaign awareness was greater among those ages 21–24. | | |
|  |  | ***(Outcome)*** | ***CA vs NCA*** | | |
|  |  | 4.Knowledge | 🢬 Campaign awareness was not significantly associated mental health related knowledge. | | |
|  |  | 5.Attitudes | 🢬 Campaign awareness was not associated with endorsement of negative stereotypes. | | |
|  |  | 6. Stigma -Desire for social distance | ⬄ Campaign awareness was associated with willingness to work closely with someone who has as serious mental illness but not moving next door to or socialising with such a person. | | |
| Collins, 2020 [42] | Cross-sectional Survey  Ages 14-24,  Los Angeles, USA,  n=1043  2019 | 1. Exposure - Campaign awareness | 75% of respondents were aware of the campaign^[[7]](#footnote-8)^ | | |
|  |  | ***(Outcome)*** | ***CA vs NCA*** | | |
|  |  | 2.Knowledge | 🢬 Campaign awareness was not significantly associated mental health related knowledge. | | |
|  |  | 3.Attitudes | ⬄🡫 Campaign awareness was associated with greater endorsement of 1 negative stereotype (that those who have had a mental illness will never contribute much to society). No other significant difference in attitudes. | | |
|  |  | 4. Stigma -Desire for social distance | 🢬 Campaign awareness was not associated with desire for social distance. | | |
| Collins, 2022a [43] | Cross-sectional Survey  Ages 18 and over,  Los Angeles, USA,  n=1002  2021 | 1. Exposure - Campaign awareness | 37% of respondents were campaign aware. | | |
|  |  | 2.Reach | Those of Spanish language–preferring Hispanic adults were more likely to be exposed to the campaign than other backgrounds. Those of Asian descent were less likely to report exposure than those from other backgrounds. 18–25-year-olds were more likely to be exposed than other age groups. Those with a high school degree or less were more likely to be exposed than more educated residents. Those with lower incomes were more likely to be exposed than higher incomes. | | |
|  |  | ***(Outcome)*** | ***CA vs NCA*** | | |
|  |  | 3.Attitudes | ⬄🡫 Campaign awareness was associated with greater endorsement of 2 negative stereotypes (that a person with a mental illness is a danger to society and that people who have had a mental illness are never going to be able to contribute much to society). No other significant difference in attitudes. | | |
|  |  | 4. Stigma -Desire for social distance | 🢬 Awareness was not associated with desire for social distance. | | |
|  |  | 5. Behaviour change – Help-seeking | 🡩Use of the website was 4x more likely in those exposed to the campaign compared with those who weren’t. | | |
| Collins, 2022b [44] | Cross-sectional Survey  Ages 18 and over,  Los Angeles, USA,  n=1010 (2020) n=1002 (2021)  2020-2021 | 1. Exposure - Campaign awareness | In 2020, 52% were campaign aware and in 2021, 37% were aware. | | |
|  |  | 2.Reach | Hispanic residents were more likely than adults from other racial, ethnic, or language backgrounds to have been exposed to the campaign. 18–25-year-olds were more likely to be exposed than other age groups. Men equally as likely to be exposed as women. | | |
|  |  | ***(Outcome)*** | ***CA vs NCA*** | | |
|  |  | 3. Stigma -Desire for social distance | 🡩 In 2021, awareness was associated with less desire for social distance but not in 2020. | | |
|  |  | 4. Behaviour change – Help-seeking | 🡩 Use of website was 3x more likely in those exposed to the campaign in 2020, and 5x more likely in those exposed to the campaign in 2021, compared with those who weren’t. Use of Help Line was 5x more likely in campaign exposed individuals in 2020 and 3x more likely in 2021. | | |
| Collins, 2022c [45] | Cross-sectional Survey  Ages 14 and over,  Los Angeles, USA,  n=1250  2022 | 1. Exposure - Campaign awareness | 27% of youth (ages 14-25) and 12% of adults (ages ≥26) were aware of campaign. | | |
|  |  | 2.Reach | Hispanic youths and adults were more likely than youth and adults from other racial, ethnic, or language backgrounds to have been exposed to the campaign. For adults, those identifying as male were more likely to be reached than those identifying as female. | | |
|  |  | ***(Outcome)*** | ***CA vs NCA*** | | |
|  |  | 3. Attitudes | ⬄🡫 Youth exposed were more likely to agree that they would delay seeking treatment for a mental health problem for fear of others finding out about their condition but no other changes in attitudes. No difference in attitudes for adults. | | |
|  |  | 4. Stigma -Desire for social distance | ⬄ Campaign awareness was associated with less desire for social distance in adults but was not for youths. | | |
|  |  | 5. Behaviour change – Help-seeking | 🡩 Campaign exposed youth were 4x more likely to report using the helpline than those who weren’t exposed. No reported use of the helpline among adults. | | |
| **Act-Belong-Commit campaign and related campaigns** | | | | | |
| Drane, 2022 [46] | Cross-sectional survey  General public, Australia, n=1200  2018-2019 | 1. Exposure - Campaign awareness | 80% were campaign aware. | | |
|  |  | ***(Outcome)*** | ***CA vs NCA*** | | |
|  |  | 2. Behaviour change – Help-seeking intentions | 🡩 Amongst those aware of the campaign, 8% stated that the campaign prompted them to seek information and 4% stated that the campaign prompted them to seek help for a mental health problem. | | |
| Santini, 2022 [47] | Cross-sectional survey  Ages 18 and over, Denmark, n=3015  2019-2021 | 1. Exposure - Campaign awareness | 12% were aware of the campaign. | | |
|  |  | ***(Outcome)*** | ***CA vs NCA*** | | |
|  |  | 2. Knowledge | 🡩 Of those that were aware, 78.4% reported that the campaign gave them new knowledge about mental health. | | |
|  |  | 3. Behaviour change – Activity to enhance mental health | 🡩 Of those that were aware, 16.2% said that they did something actively to enhance their mental health because of hearing about the campaign. | | |
| **In One Voice campaign** | | | | | |
| Livingston, 2013 [48] | Surveys pre and post campaign  Ages 13-25, British Columbia, Canada, n=403 (pre) n=403 (post)  2012 | 1. Exposure - Campaign awareness | 25% were aware of the campaign. | | |
|  |  | 1. Exposure - Views | 1 month after the campaign number of page views increased by 201% to 364,792 views. | | |
|  |  | 3. Low engagement | The campaign was ‘liked’ on Facebook by 1,640 people. | | |
|  |  | ***(Outcome)*** | ***Baseline vs Follow-up*** | | ***CA vs NCA*** |
|  |  | 3. Knowledge | 🢬 Mental health knowledge did not change 2 months after the campaign. | | NR |
|  |  | 4. Attitudes | 🢬 Attitudes did not change. | | NR |
|  |  | 5. Stigma -Desire for social distance | ⬄ One item (willingness to invite someone with a mental illness to their home) on the desire for social distance outcome measurement showed significant improvement. | | NR |
|  |  | 6. Behaviour change – Help-seeking intention and aware of how to access care | 🢬 No significant change over 2 months | | 🡩 Awareness was associated with better intended behaviours across all 3 items; discuss mental health issues with others, learn about sings/symptoms and learn about accessing mental health services |
| Livingston, 2014 [49] | Surveys over multi cross-sectional waves  Ages 13-25, British Columbia, Canada,  n=403 (2012) n=438 (2013)  2012-2013 | 1. Exposure - Campaign awareness | 49% were aware of the campaign in 2013 vs 25% in 2012. | | |
|  |  | 2. Exposure - Views | Page views increased from 26,156 pre campaign to 71,982 56 days post campaign and 96,106 1-year post campaign. | | |
|  |  | ***(Outcome)*** | ***Baseline vs Follow-up*** | | |
|  |  | 3. Knowledge | 🢬 Mental health knowledge did not change 1 year after the campaign. | | |
|  |  | 4. Attitudes | 🡩 Attitudes towards mental health improved 1 year after the campaign. | | |
|  |  | 5. Stigma -Desire for social distance | 🡩 Desire for social distance decreased 1 year after the campaign. | | |
|  |  | 6. Behaviour change – Aware of how to access care | ⬄ The only help-seeking behaviours to change 1 year after the campaign was respondents indicated that they had made a personal effort to learn more about how to access mental health information or service. | | |
| **WhatMakesUs and Action Minded campaigns** | | | | | |
| Public Goods People, 2019 [55] | Cross-sectional survey pre and post campaign and social media metrics  Ages 18-65, Colorado, Hawaii, Georgia, Oregon, Washington, Northern California, Southern California and Mid-Atlantic States, USA n= 4080 n= 2039 (pre) n= 2014 (post)    2018-2019 | 1. Exposure - Campaign awareness | 49.4% of respondents reported campaign awareness at follow-up. | | |
|  |  | 2. Exposure – Impressions and views | 402,064 video views. 23,766,340 impressions | | |
|  |  | 3. Reach | Of those reporting campaign awareness 76.2% reported ever having a mental health condition. | | |
|  |  | 4. Low engagement | 311,228 engagements. | | |
|  |  | ***(Outcome)*** | ***Baseline vs follow-up*** | ***CA vs NCA*** | |
|  |  | 5. Knowledge (MAKS) | 🡩 Mental health knowledge showed significant improvement after 1 year | 4. 🡩 A significantly higher proportion of respondents in the campaign aware group reported improved mental health knowledge compared to those in the not campaign aware group. | |
|  |  | 6. Attitudes (CAMI) | 🡩 Attitudes towards people with mental health conditions showed significant improvement after 1 year. | 🡩 Significantly greater proportion of campaign aware respondents reported improved attitudes compared to not campaign aware respondents. | |
|  |  | 7. Stigma -Desire for social distance (RIBS) | 🢬 Respondents desire for social distance was not significantly different after 1 year. | 🡩 Significantly reduced desire for social distance in the campaign aware group compared to the not campaign aware group. | |
|  |  | 8. Behaviour change - Activity to enhance mental health | 🢬 No difference for participants taking steps to improve their mental health in the preceding 6 months. | 🡩 Greater proportion of respondents with campaign awareness took steps to improve their own mental health or provide support to someone else’s in the preceding 6 months than those without campaign awareness. | |
| Diouf, 2022 [51] | Cross-sectional survey pre and post campaign conducted in intervention and control areas.  Ages 18-65,  Omaha Council Bluffs Iowa metropolitan region n= 868 n= 466 (pre), n= 402 (post)  Intervention (campaign exposed) = Omaha and Council Bluffs area n= 476 n= 246 (pre), n= 230 (post)  Control (not campaign exposed)= Iowa region n= 392 n= 220 (pre), n= 172 (post)  2020-2021 | 1. Exposure - Campaign awareness | 30% of respondents within the intervention group reported campaign awareness | | |
|  |  | ***(Outcome)*** | ***Baseline vs follow-up (after 10 months)*** | ***CA vs NCA (follow-up sample)*** | |
|  |  | 2. Knowledge | ⬄ Intervention: Significant improvement in medication knowledge, but non-significant improvement in therapy and counselling knowledge  🡫 Control: significant decrease in mental health knowledge. | 🢬 Non-significant higher levels of mental health knowledge in campaign aware group. | |
|  |  | 3. Attitudes | 🢬 Intervention: non-significant improvement in attitudes after 10 months.  🢬 Control: non-significant decrease in attitudes after 10 months. | 🡩 Campaign aware group had significantly less stigmatising attitudes towards someone with a mental health condition. | |
|  |  | 4. Stigma -Desire for social distance | 🡩 Intervention: significantly reduced desire for social distance after 10 months.  🢬 Control: non-significant increased desire for social distance after 10 months. | 🡩 Significantly more individuals in campaign aware group had reduced desire for social distance. | |
|  |  | 5. Behaviour change - Activity to enhance mental health | 🢬 Intervention: A non-significant increase in steps taken to improve mental health in the preceding 6 months.  🢬 Control: No clear change in help-seeking behaviour | 🡩 Significantly more campaign aware respondents took steps to improve their own mental health or provide support to someone else’s in the preceding 6 months than those without campaign awareness. | |
| Alvarado-Torres, 2023 [52] | Cross-sectional survey and social media metrics  Ages 18-65,  3 counties in Nebraska, USA and 1 in Iowa, USA n= 334  2021-2022 | 1. Exposure - Campaign awareness | 53% were campaign aware. | | |
|  |  | 2. Exposure – impressions and views | Total of 2,558,291 impressions, Facebook accounted for 1,838,300 whilst Instagram accounted for 719,991. Average daily campaign views were 1,745, Facebook averaging 1,288 and Instagram averaging 457. Total video views through social media were 1,262,871. Facebook accounted for 123,999 video views and Instagram 2,288. | | |
|  |  | 3. Reach | Most campaign aware respondents were non-Hispanic White, men and aged between 25 and 34. | | |
|  |  | 4. Low and medium engagement | Engagements were measured as likes, comments, shares, video views or post clicks. Total number of engagements was 27,053. Facebooked received 23,763 engagements whilst Instagram received 3,290. | | |
|  |  | ***(Outcome)*** | ***CA vs NCA*** | | |
|  |  | 5. Knowledge | ⬄ Significant improvement in therapy and counselling knowledge, but non-significant improvement in medication knowledge in the campaign aware group. | | |
|  |  | 6. Attitudes | 🢬 Campaign aware group had non-significantly lower stigmatising attitudes compared to not campaign aware respondents. | | |
|  |  | 7. Stigma -Desire for social distance | ⬄ Campaign awareness was associated with living with, working with and being close friends with a person with a mental health condition but not willingness to live with, work with and be close friends with a person with a mental health condition. | | |
|  |  | 8. Behaviour change - Activity to enhance mental health | 🡩 A significantly higher frequency of campaign aware respondents reported taking steps to improve their mental health in the last 6 months compared to those not-campaign aware. | | |
| **Other campaigns** | | | | | |
| Hann, 2016 [53] | Cross-sectional survey  General public,  Kent UK,  n=53  2014-2016 | 1. Exposure - Impressions | 246,255 X (formerly Twitter) impressions. | | |
|  |  | 1. Reach | 57% of viewers of this YouTube video content were female. | | |
|  |  | 1. Low engagement | There was an increase from 246 Facebook likes in February 2014 to 758 likes in January 2016. On average, the Liveitwell Facebook page attracted 22.2 ‘likes’ per month. The most viewed video on the YouTube channel was Wheel of Wellbeing video, with 11,993 views, 36 likes. 440 Tweet likes from January 2014 to December 2015. | | |
|  |  | 1. Medium engagement | 2,563 Tweets and 1,231 Retweets. 100 shares of videos on YouTube. | | |
| Hahn, 2023 [54] | Surveys over multi cross-sectional waves  Ages 18 and over,  UK,  n=2000-3000 (per each survey – 9 waves)  Total n= 20,435  2019-2022 | ***(Outcome)*** | ***Baseline vs Follow-up*** | | ***CA vs NCA (n=13,178 of sample)*** |
|  |  | 1. Knowledge (MHL-REC and MHL-ACT scale)^[[8]](#footnote-9)^ | ⬄ Small improvements in all knowledge items between September 2019 and March 2020. Beyond March 2020 changes reversed and knowledge declined from 2019. | | ⬄ Those aware of the campaign showed a small increase in symptom management for depression and anxiety but not recognition of these symptoms. |
|  |  | 1. Stigma -Desire for social distance (RIBS) | 🡫 Stigma related to mental health disorders declined from September 2019 to March 2022. | | 🡩 Campaign awareness associated with lower desire for social distance |
|  |  | 1. Behaviour change - Help-seeking intentions (MHLS and SRAHPS)^[[9]](#footnote-10)^ | 🡫 Help-seeking self-efficacy (MHLS) scores and psychological wellbeing self-efficacy (SRAHPS) showed a decline from September 2019 to March 2022. | | ⬄ Campaign awareness was associated with help-seeking self-efficacy but not psychological wellbeing self-efficacy. |
| Hansson, 2016 [55] | Surveys pre and post campaign  Sweden,  n=2053-2317 (each year for national surveys) and n=657-1153  (2009-2014 each year for regional surveys)  2009-2014 | ***(Outcome)*** | ***Baseline vs Follow-up*** | | |
|  |  | 1. Knowledge (MAKS | 🡩 Mental health literacy improved significantly in the campaign regions between 2009 and 2014. | | |
|  |  | 1. Attitudes (CAMI) | 🡩 Attitudes toward mental illness significantly improved in the campaign regions between 2009 and 2014. | | |
|  |  | 1. Stigma -Desire for social distance (RIBS) | 🡩 Desire for social distance significantly reduced in the campaign regions between 2009 and 2014. | | |
| Booth, 2018 [24] | Multi cross-sectional waves  Ages 10-24,  Ontario, Canada,  n= 2,576,630 (2006) to n= 2,564,097 (2015)  2006-2015 | 1. Medium engagement | 78,520,284 interactions (retweets use of hashtags) | | |
|  |  | ***(Outcome)*** | 1. ***Baseline vs Follow-up*** | | |
|  |  | 1. Behaviour change- Help seeking | 1. 🡩 The campaign was associated with a statistically significant increase in the mental health visits rates, especially for adolescent females, but young adults exhibited an immediate and slight drop post campaign in most outpatient mental health visits, followed by a moderate increase or plateauing of rates. | | |
| Zenone, 2020 [56] | Surveys pre and post campaign and social media metrics  Ages 12-17 years in 2 schools in British Columbia, Canada  n=298  NR | 1. Exposure - Campaign awareness | In school 1, 26% of students were aware of the campaign, 46% of students in school 2 were aware. | | |
|  |  | 1. Exposure - Views | On Snapchat, approximately 160,000+ views and sent 7,325 swipe-ups to the Foundry website. Instagram, resulted in 170,000+ views and approximately, 2,467 clicks were sent to the Foundry website. Google analytics showed a 70% increase in new user traffic to foundrybc.ca in British Columbia when compared with the previous 3 months before the campaign (18,881 vs. 11,126). | | |
| Collins, 2019 [57] | Cross-sectional survey  Ages 18 and over,  California, USA  n=1954  2014-2016 | 1. Exposure - Campaign awareness | 27.6% of respondents were exposed to the campaign. | | |
|  |  | ***(Outcome)*** | ***CA vs NCA*** | | |
|  |  | 2. Knowledge | 🢬 Awareness of the campaign was not associated with mental health knowledge. | | |
|  |  | 3. Attitudes | ⬄ Awareness was associated with more positive recovery beliefs but not with negative beliefs about mental illness, treatment attitudes, intention to conceal a hypothetical future mental health problem, awareness of public stigma or mental health knowledge and advocacy. | | |
|  |  | 4. Behaviour change - Help-seeking | 🡩 There was a significant positive association between campaign awareness and seeing a mental health practitioner in the last 12 months (odds ratio [OR] = 1.82; 95%CI = 1.17, 2.83. | | |
| Thompson, 2021 [58] | Surveys pre and post campaign  Ages 11-18 Students across 22 schools Midwestern County, USA,  n=11478 (pre and post)  2017-2018 | ***(Outcome)*** | ***Baseline vs Follow-up*** | | |
|  |  | 1. Attitudes | ⬄ Significant overall improvements in student stigma but students with African American ethnicity reported less improvement than those of White ethnicity. | | |
|  |  | 1. Behaviour change - Help-seeking intentions | ⬄ Significant overall improvements in help-seeking attitudes but students with African American ethnicity reported less improvement than those of White ethnicity. | | |
| Coughlan, 2021 [59] | Social media metrics  General public, Ages 18 and over  2019-2020 | 1. Exposure - Views | Animations were viewed 15,848 times. Most views occurred during the period of the social media ad campaign. Facebook views accounted for almost two-thirds of all views. 10,437 views through Facebook, 1949 views through Twitter, 3462 views through Youtube. Low rates of link clicks on Facebook (n=240). | | |
|  |  | 1. Reach | 55.1% of those who viewed the animations on Facebook were aged 18-24 years, 39.9% were aged 13-17 years, and the remaining 5.0% were aged 25 years or over. There were higher rates of female viewers than males across all animations and age ranges but the Being Bullied animation, there were more male views. | | |

1. If a validated survey was used to measure the outcome this will be described in brackets following the outcome, otherwise the outcome was measured using bespoke survey questions. [↑](#footnote-ref-2)
2. 🢬: no change in outcome, ⬄: mixed outcomes, 🡩: improvement in outcome, 🡫: negative outcome [↑](#footnote-ref-3)
3. Campaign aware vs Not Campaign Aware [↑](#footnote-ref-4)
4. Mental Health Knowledge Schedule (MAKS) [↑](#footnote-ref-5)
5. Community Attitudes toward the Mentally Ill (CAMI) [↑](#footnote-ref-6)
6. Reported and Intended Behaviour Scale (RIBS) [↑](#footnote-ref-7)
7. Measure of awareness was significantly different from 2019 and is why awareness is much higher than 20% reported in 2019. In this year it incorporated awareness of any part of the campaign not just campaign tittle. [↑](#footnote-ref-8)
8. MHL-REC: Mental Health Literacy—Knowledge for Recognition scale; MHL-ACT: Mental Health Literacy—Knowledge for Management scale. [↑](#footnote-ref-9)
9. MHLS: Mental Health Literacy Scale; SRAHPS: Self-Rated Abilities for Health Practices Scal. [↑](#footnote-ref-10)
