# Supplementary material for: The Effectiveness of Social Media Campaigns in Improving Knowledge and Attitudes Toward Mental Health and Help-Seeking in High-Income Countries: Scoping Review
Source: J Med Internet Res. 2025 May 23;27:e68124. doi: 10.2196/68124 (PMC12144482; doi:10.2196/68124)
Supplement: Multimedia Appendix 4 [file jmir_v27i1e68124_app4.docx]

**Knowledge**

In the Time to Change campaign 40% (2/5) of articles assessing knowledge over time reported that knowledge improved [1, 2] with 60% (3/5) reporting no changes [3-5], but all the articles comparing knowledge by campaign awareness (3/3, 100%) found campaign awareness was associated with improved knowledge [3-5].

In the Act-Belong-Commit and related campaigns, one article (1/2, 50%) assessed knowledge and found campaign awareness was associated with improved knowledge [6].

In the WhatMakesUs and Action Minded campaigns, of the three articles that assessed knowledge over time one (1/2, 50%) found an improvement in knowledge [7] and one found mixed findings [8]; one article found campaign awareness improved knowledge (1/3, 33%) [7] but one found no significant change [8] and one found mixed results [9].

In the WhyWeRise and In One Voice campaigns all the articles assessing knowledge found no difference in knowledge between those who were campaign and those who were not or over time.

**Attitudes**

In the Time to Change campaign 40% (2/5) of articles assessing attitudes over time reported that knowledge improved [1, 2] with 60% (3/5) reporting no changes [3-5]. One article (1/3, 33%) found campaign awareness was associated with more positive attitudes [5], one found no significant difference [4] and one found mixed findings, with no significant change found for the item about whether people with mental health problems should not be given any responsibility [3].

In the WhatMakesUs and Action Minded campaigns, of the two articles that assessed attitudes over time one (1/2, 50%) found an improvement in attitudes [7] and one found no differences [8]; two articles found campaign awareness improved attitudes (2/3, 67%) [7, 8] but one found no significant change [9].

In the One Voice campaigns, one article (1/2, 50%) that assessed attitudes over time found it improved attitudes [10] but the other found no change [11].

In the earliest evaluation of the WhyWeRise campaigns in 2018, there was no association between campaign awareness and attitudes [12]. In 2019, they found mixed results, with one negative stereotype endorsed, that those who have had a mental illness will never contribute much to society [13]. In 2021, they found campaign awareness was associated with endorsement of two negative stereotypes, that a person with a mental illness is a danger to society and that people who have had a mental illness are never going to be able to contribute much to society [14]. In the latest evaluation in 2022, it was found that youth exposed to campaign materials reported a desire to delay seeking mental health treatment out of fear of others finding out but no other attitudes differed and no attitudes differed for adults [15].

**Stigma - Desire for social distance**

In the Time to Change campaign 40% (2/5) of articles assessing desire for social distance over time reported that this improved [1, 2], with 40% (3/5) reporting no changes [4, 5] and one (1/5, 20%) finding that only the item about being willing to live with someone with a mental health problem had increased over the campaign [3]. All the articles comparing desire for social distance by campaign awareness (3/3, 100%) found campaign awareness was associated with reduced desire for social distance [3-5].

In the WhatMakesUs and Action Minded campaigns, of the two articles that assessed desire for social distance over time one (1/2, 50%) found a reduction in desire for social distance [8] and one found no differences [7]; two articles found campaign awareness reduced desire for social distance (2/3, 67%) [7, 8]. One article reporting mixed findings found that actual behaviours related to social distancing, such as living with a person with a mental health condition, showed significant improvement, whereas intended behaviours, such as willingness to live with a person with a mental health condition, showed non-significant improvement [9].

In the One Voice campaigns, one article (1/2, 50%) that assessed desire for social distance over time, found this improved [10] but the other found mixed findings, with only item about willingness to invite someone with a mental illness to their home significantly improving [11].

In the earliest evaluation of the WhyWeRise campaigns in 2018, campaign awareness was associated with willingness to work closely with someone who has as serious mental illness but not moving next door to or socialising with such a person [12]. In 2019 and 2020, they found no association between campaign awareness and desire for social distance [13, 16]. In 2021, campaign awareness was associated with reduced desire for social distance [14]. In the latest evaluation in 2022, it was found that youth exposed to campaign materials reported a desire to delay seeking mental health treatment out of fear of others finding out but no other attitudes differed and no attitudes differed for adults [15].

**Behaviour change**

In the Time to Change campaign one article (1/1, 100%) found that help-seeking intentions over time declined but this article also found that campaign awareness was associated with improved help-seeking intentions [17].

In the Act-Belong-Commit and related campaigns, the two articles (2/2, 100%) assessing behaviour change found that campaign awareness was associated with improved help-seeking intentions [18] and activities to enhance mental health [6].

In the WhatMakesUs and Action Minded campaigns, of the two articles that assessed behaviour change over time both found no significant difference in activities to enhance mental health [7, 8]. All three articles (3/3, 100%) assessing behaviour change found that campaign awareness was associated with improved activities to enhance mental health [7-9].

In the One Voice campaigns, one article (1/2, 50%) that assessed behaviour change over time, found no difference [11] but the other found mixed findings, with only item making an effort to learn about accessing mental health services being the only one to show significant improvement [10].

In the WhyWeRise campaigns all three articles (3/3, 100%) assessing behaviour change found campaign awareness was associated with help-seeking, primarily use of helplines and websites provided by the campaign[14-16]. In 2020, helpline use was 5 times more likely in those exposed to the campaign, and 3 times more likely in those exposed to the campaign in 2021 [16]. In 2022, campaign exposed youth were 4 times more likely to report using the helpline than those who were not exposed, but adults did not use the helpline [15].

**References**

1. Henderson C, Potts L, Robinson EJ. Mental illness stigma after a decade of Time to Change England: inequalities as targets for further improvement. European journal of public health. 2020;30(3):497-503.

2. Henderson C, Robinson E, Evans‐Lacko S, Corker E, Rebollo‐Mesa I, Rose D, et al. Public knowledge, attitudes, social distance and reported contact regarding people with mental illness 2009–2015. Acta Psychiatrica Scandinavica. 2016;134:23-33.

3. Evans-Lacko S, Malcolm E, West K, Rose D, London J, Rusch N, et al. Influence of Time to Change's social marketing interventions on stigma in England 2009-2011. British Journal of Psychiatry. 2013 2013;202:S77-S88. PMID: rayyan-339582510.

4. González-Sanguino C, Potts, L.C., Milenova, M. Henderson, C. Time to Change’s social marketing campaign for a new target population: results from 2017 to 2019. BMC psychiatry. 2019;19:1-11.

5. Sampogna G, Bakolis I, Evans-Lacko S, Robinson E, Thornicroft G, Henderson C. The impact of social marketing campaigns on reducing mental health stigma: Results from the 2009–2014 Time to Change programme. European Psychiatry. 2017;40:116-22.

6. Santini ZI, Nelausen MK, Kusier AO, Hinrichsen C, Schou-Juul F, Madsen KR, et al. Impact evaluation of the “ABCs of Mental Health” in Denmark and the role of mental health-promoting beliefs and actions. Mental Health and Social Inclusion. 2022;26(3):271-91.

7. The Public Good Projects. Action Minded: Reducing Mental Health Stigma Using Digital Media Campaigns. 2019.

8. Diouf F, Lemley B, Barth C, Goldbarg J, Helgenberger S, Grimm B, et al. Mental health stigma reduction in the Midwestern United States: Evidence from a digital campaign using a collective impact model. Journal of Community Health. 2022;47(6):924-31.

9. Alvarado-Torres R, Dunn Silesky M, Helgenberger S, Anderson A, Granillo C, Nared T, et al. Evaluation of a digital media campaign for reducing mental health stigma. Health Education Journal. 2023:00178969231215761.

10. Livingston JD, Cianfrone M, Korf-Uzan K, Coniglio C. Another time point, a different story: one year effects of a social media intervention on the attitudes of young people towards mental health issues. Social psychiatry and psychiatric epidemiology. 2014;49:985-90.

11. Livingston JD, Tugwell A, Korf-Uzan K, Cianfrone M, Coniglio C. Evaluation of a campaign to improve awareness and attitudes of young people towards mental health issues. Social psychiatry and psychiatric epidemiology. 2013;48(6):965-73.

12. Collins RL, Eberhart NK, Marcellino W, Davis L, Roth E. Evaluation of Los Angeles County's Mental Health Community Engagement Campaign. Santa Monica, CA: RAND Corporation; 2018.

13. Collins RL, Eberhart NK, Seelam R, De Guttry R, Mizel ML. 2019 Evaluation of Los Angeles County's WhyWeRise Mental Health Campaign. 2020.

14. Collins RL, Eberhart NK, Roth E. Evaluation of Los Angeles County's 2021 WhyWeRise Mental Health Campaign. 2022.

15. Collins RL, Eberhart NK, Estrada-Darley I, Roth E. Evaluation of Los Angeles County's L.A. Dodgers 2022 Mental Health Campaign. 2022.

16. Collins RL, Eberhart NK, Roth E, Estrada-Darley I. Evaluation of Los Angeles County Department of Mental Health's 2020-2021 L.A. Dodgers Mental Health Campaign. Santa Monica, CA: RAND Corporation; 2022.

17. Henderson C, Robinson E, Evans-Lacko S, Thornicroft G. Relationships between anti-stigma programme awareness, disclosure comfort and intended help-seeking regarding a mental health problem. British Journal of Psychiatry. 2017 2017;211(5):316-22. PMID: rayyan-339582511.

18. Drane CF, Jalleh G, Lin C, Donovan RJ. Impact of the Act‐Belong‐Commit campaign on mental health help‐seeking behaviour. Health Promotion Journal of Australia. 2023;34(1):232-6.
