# Supplementary figures and images for: The Effectiveness of Social Media Campaigns in Improving Knowledge and Attitudes Toward Mental Health and Help-Seeking in High-Income Countries: Scoping Review
Source: J Med Internet Res. 2025 May 23;27:e68124. doi: 10.2196/68124 (PMC12144482; doi:10.2196/68124)

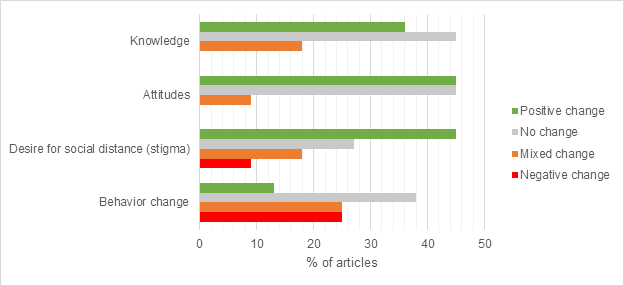

Supplement: Multimedia Appendix 5 [file jmir_v27i1e68124_app5.png]

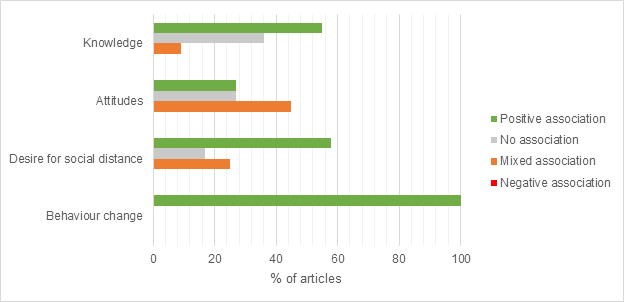

Supplement: Multimedia Appendix 6 [file jmir_v27i1e68124_app6.png]
